# New Medical Journals

**Published:** 1850-11

**Authors:** 


### ARTICLE VII.

### NEW MEDICAL JOURNALS.

We have received a new Medical Journal published in the city of New York, under the title of the *New York Register of Medicine and Pharmacy*. It is published semi-monthly, at one dollar per annum in advance. The editor is C. D. Griswold, M.D., who, to judge from the numbers already received, will conduct it with spirit and ability. The mechanical execution of the Journal is remarkably good.

We also gave notice some time since, of the prospectus of a new medical periodical to be issued at Keokuk, in Iowa. The first number has come to hand under the style of *The Western Medico-Chirurgical Journal*, edited by J. F. Sandford, M.D., and S. G. Armor, M.D., Professors in the Medical Department of the University of Iowa. It is issued monthly, (each No containing thirty-two pages) at two dollars per annum. The present No. contains some very interesting articles, and its appearance is quite creditable to the *Hawkeye* typographers.

We wish both of our new allies better fortune than usually falls to the lot of Medical Editors and Periodicals. M.

